# CIRSE Standards of Practice on Endovascular Treatment of Acute Pulmonary Embolism

**DOI:** 10.1007/s00270-025-04312-3

**Published:** 2026-01-05

**Authors:** Juan J. Ciampi-Dopazo, José A. Guirola, John Moriarty, Raman Uberoi, Dimitrios Tsetis, Corrado Ini’, Antonio Basile

**Affiliations:** 1 Hospital Universitario Virgen de Las Nieves, Granada, Spain; 2 Hospital Clínico Lozano Blesa, Saragoza, Spain; 3 UCLA Medical Center, Los Angeles, United States; 4 John Radcliff Hospital, Oxford, England; 5 University Hospital Heraklion, Heraklion, Greece; 6 Department of Medical Sciences, Surgican and Advanced Technologies, Policlinico Universitario G. Rodolico, Catania, Italy; 7 Azienda Ospedaliera Universitaria Policlinico Rodolico-San Marco, UOC Radiologia 1, Catania, Italy

**Keywords:** Thrombectomy, Pulmonary embolism, Thrombolytic therapy, Pharmacomechanical thrombectomy, Embolectomy

## Abstract

**Purpose:**

This CIRSE Standards of Practice document aims to provide comprehensive standards for the endovascular treatment of acute pulmonary embolism and includes recommendations for the imaging diagnosis, surveillance, intervention indications and endovascular treatments.

**Methods:**

The CIRSE Standards of Practice Committee established a writing group of six internationally recognised interventional radiologists with expertise in pulmonary embolism interventions and one research assistant (C.I). The group conducted a pragmatic evidence-based PubMed search for relevant English-language reports on human subjects up to early 2025. The final recommendations are consensus-based.

**Results:**

Endovascular treatment of pulmonary embolism is highly successful with low complication rates. For acute pulmonary thromboembolic disease, catheter-directed thrombolysis and mechanical thrombectomy are options for patients with intermediate–high-risk and high-risk pulmonary embolism, especially when systemic fibrinolysis fails or is contraindicated.

**Conclusions:**

Endovascular therapy for acute pulmonary embolism is both safe and effective. This best practice document emphasises early diagnosis, appropriate patient selection and timely intervention.

## Introduction

The CIRSE Standards of Practice (SOP) Committee established a writing group that was tasked with producing up-to-date recommendations for the performance of endovascular treatment for acute pulmonary thrombo-embolism (APTE). This SOP is neither a clinical practice guideline nor a systematic review of the literature. CIRSE SOP documents are not intended to impose a standard of clinical patient care but rather recommend a reasonable approach to and best practices for the performance of endovascular treatment in such conditions. Institutions should regularly review their internal procedures for development and improvement, considering international guidance, local resources and having a framework for regular governance review of the service. A summary of key recommendations for the acute treatment of APTE can be found in Table 1.

**Table 1:** Summary of key recommendations

Diagnosis—Computed tomography pulmonary angiography (CTPA)
Recommended first-line imaging study for suspected APTE due to its high accuracy and ability to visualise pulmonary arteries to subsegmental level
In patients with persistent or new-onset dyspnoea or exercise limitation following APTE, further diagnostic evaluation to assess for CPTEH/CPTED is recommended. Referral to a pulmonary hypertension/CPTEH centre is recommended after considering the results of echocardiography, BNP/NT-proBNP and/or cardiopulmonary exercise testing. It should not be the initial imaging diagnostic test for CPTED. Right heart catheterisation (RHC) may be necessary to confirm the diagnosis and assess the severity of disease
Risk stratification of patients for endovascular treatment in acute pulmonary thromboembolism
High-risk PTE patients
Patients with haemodynamic instability (e.g. cardiac arrest, obstructive shock (systolic BP < 90 mmHg), persistent hypotension (systolic BP < 90 mmHg or a systolic BP drop ≥ 40 mmHg for > 15 min not caused by other conditions such as new arrhythmia, hypovolemia or sepsis)
Intermediate-to-high-risk APTE patients
Patients with haemodynamic stability but with:
Right ventricular
Dysfunction assessed by either transthoracic echocardiography (TTE):
Dilated RV with basal RV/LV Ratio ≥ 1.0
Decreased Tricuspid Annular Plane Systolic Excursion (TAPSE) < 16 mm
Peak systolic (S’): < 9.5 cm/s
Or by CTPA:
Enlarged right ventricle:
RV/LV ratio ≥ 0.9 is an independent predictor of adverse in-hospital outcomes
Contrast reflux into the IVC:
The presence of contrast reflux into the IVC on CTPA is another sign of right heart strain and is associated with an adverse prognosis
Pulmonary Embolism Severity Index (PESI) score of Class III-V or a simplified PESI (sPESI) score of ≥ 1
Elevated cardiac biomarkers:
Elevated cardiac troponins
Elevated BNP or NT-proBNP levels
Specific considerations for endovascular treatment of acute pulmonary thromboembolism
Time window: 14 days or less since symptom onset and CTPA evidence of proximal APTE defined as a filling defect in at least one main or lobar PA
Advanced monitoring: medical anaesthetists or experienced intensive care doctors are crucial for patient monitoring. For patients with intermediate- or high-risk APTE, it is advised to avoid general anaesthesia
Sedation as a standard procedure is not recommended
In particular, Propofol should be avoided due to the increased risk of complications
Intubation and mechanical ventilation should only be used if absolutely essential
The available treatment options are catheter-directed thrombolysis, mechanical thrombectomy or a combination of both
Venous approach: ultrasound-guided jugular or femoral vein approach
Anticoagulation should be maintained within the therapeutic range throughout the intervention, with an aPTT ratio of 1.8–2.5 or an aPTT of 60–85 s
Long sheath: It is recommended to use a long sheath (65–90 cm) to prevent cardiac or APTE injury when advancing or retrieving a catheter
Limiting factors include the size and volume of the clots, age of the clots (older clots tend to reorganise and adhere to the vessel wall) and the patient’s haemodynamic/respiratory status
Consider IVC filter placement in patients with contraindications for fibrinolysis or anticoagulation
Extracorporeal membrane oxygenation (ECMO) or venoarterial extracorporeal membrane oxygenation (V-A ECMO) may be considered for patients experiencing refractory circulatory collapse or cardiac arrest

*APTE* Acute pulmonary thromboembolism, *aPTT* activated Prothrombin time, *BNP* B-type natriuretic peptide *CI* Cardiac Index, *CPTED* Chronic pulmonary thromboembolic disease, *CPTEH* Chronic pulmonary thromboembolic hypertension, *CTPA* Computed tomography pulmonary angiography, *ECMO* Extracorporeal membrane oxygenation, *IVC* Inferior vena cava, *mPAP* mean Pulmonary artery pressure, *NYHA* New York Heart Association status, *PA* pulmonary artery, *PE* Pulmonary embolism, *PESI* Pulmonary Embolism Severity Index, *PTE* Pulmonary thromboembolism, *RHC* Right heart catheterisation, *RV* Right ventricular, *RV/LV ratio* Right ventricle/left ventricle ratio, *sPESI* simplified PESI, *TTE* transthoracic echocardiography, *TAPSE* Tricuspid Annular Plane Systolic Excursion, *V-A ECMO* Venoarterial extracorporeal membrane oxygenation

## Methods

The writing group, established by the CIRSE SOP Committee, consisted of six internationally recognised interventional radiologists with expertise in APTE interventions and one research assistant (C.I). The writing group reviewed existing literature, performing a pragmatic evidence search using PubMed to search for relevant English-language publications up to 2025.

## Background

Venous thromboembolic disease (VTE), including deep vein thrombosis (DVT) and APTE, is a common cardiovascular condition with an annual incidence of 100–200 cases per 100,000 population [1, 2]. The annual incidence of APTE ranges from 39 to 115 cases per 100,000 population with 5% of these patients presenting with massive APTE and a reported mortality rate of 58% at 3 months [3, 4]. Early diagnosis, appropriate patient selection and timely intervention are major factors in improving outcomes for patients with APTE [5, 6]. Traditionally, the mainstay of treatment for these patients has been standard anticoagulation with either systemic thrombolysis and/or surgery. Endovascular treatments have become increasingly utilised over the last 10 years and are the subject of this SOP document.

In addition to the acute complications, incomplete resolution of APTE can lead to significant sequelae, with one-third of patients demonstrating clinical features of residual post-APTE syndrome a year later [7, 8]. APTE can evolve into chronic pulmonary thromboembolic disease (CPTED) or chronic pulmonary thromboembolic hypertension (CPTEH) in 0.1–9.1% of patients within two years [9, 10].

### Clinical Presentation

Symptoms can vary widely with many patients being completely asymptomatic, some having mild symptoms of dyspnoea and some presenting with complete circulatory collapse [3, 11]. Working closely with other clinical colleagues is critical to ensure a rapid and accurate diagnosis of APTE, as well as triaging patients who require endovascular treatment: these are an essential part of developing and setting up a APTE service. This so-called pulmonary embolism response team (PERT) may include emergency doctors, anaesthetists, cardiologists, chest physicians, intensivists, cardiothoracic surgeons and of course radiology colleagues. A protocolled pathway should be developed collaboratively with these team members locally to ensure optimal outcomes for patients.

### Diagnosis

**Fig. 1:**
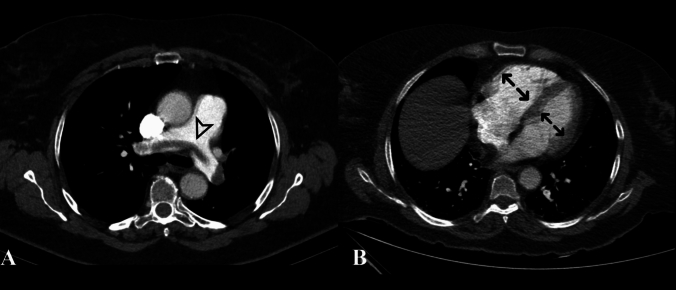
Axial CT pulmonary angiography (CTPA) demonstrating a saddle thrombus straddling the main pulmonary artery (white arrow, panel A). Panel B shows measurement of the right ventricle-to-left ventricle (RV/LV) diameter ratio (black arrows), an indicator of right ventricular strain if > 0.9

Computed tomography pulmonary angiography (CTPA) should be utilised for diagnosing APTE, even in critically unwell patients. It should be undertaken for all patients with an intermediate or high pre-test probability. The CTPA can, in addition to confirming the diagnosis, demonstrate the extent of thrombus in the pulmonary arteries and also help risk-stratify patients using the right ventricle/left ventricle ratio (RV/LV, > 0.9 abnormal) (Fig. 1). However, echocardiography should also be used to determine the presence of acute right ventricular (RV) strain/failure [3]. In patients in whom CTPA is contraindicated (such as those with severely impaired renal function defined by a creatinine clearance < 30 ml/min), ventilation/quantity of perfusion (V/Q) scanning is the test of choice (but only in stable patients and will not be available 24/7). Another option may be lower-limb compression ultrasonography which shows a DVT in 30–50% of patients with APTE. A positive finding of a proximal DVT in patients suspected of having APTE is considered sufficient to warrant anticoagulant treatment without further testing [3, 12].

### Acute Pulmonary Thromboembolism Treatment

**Fig. 2:**
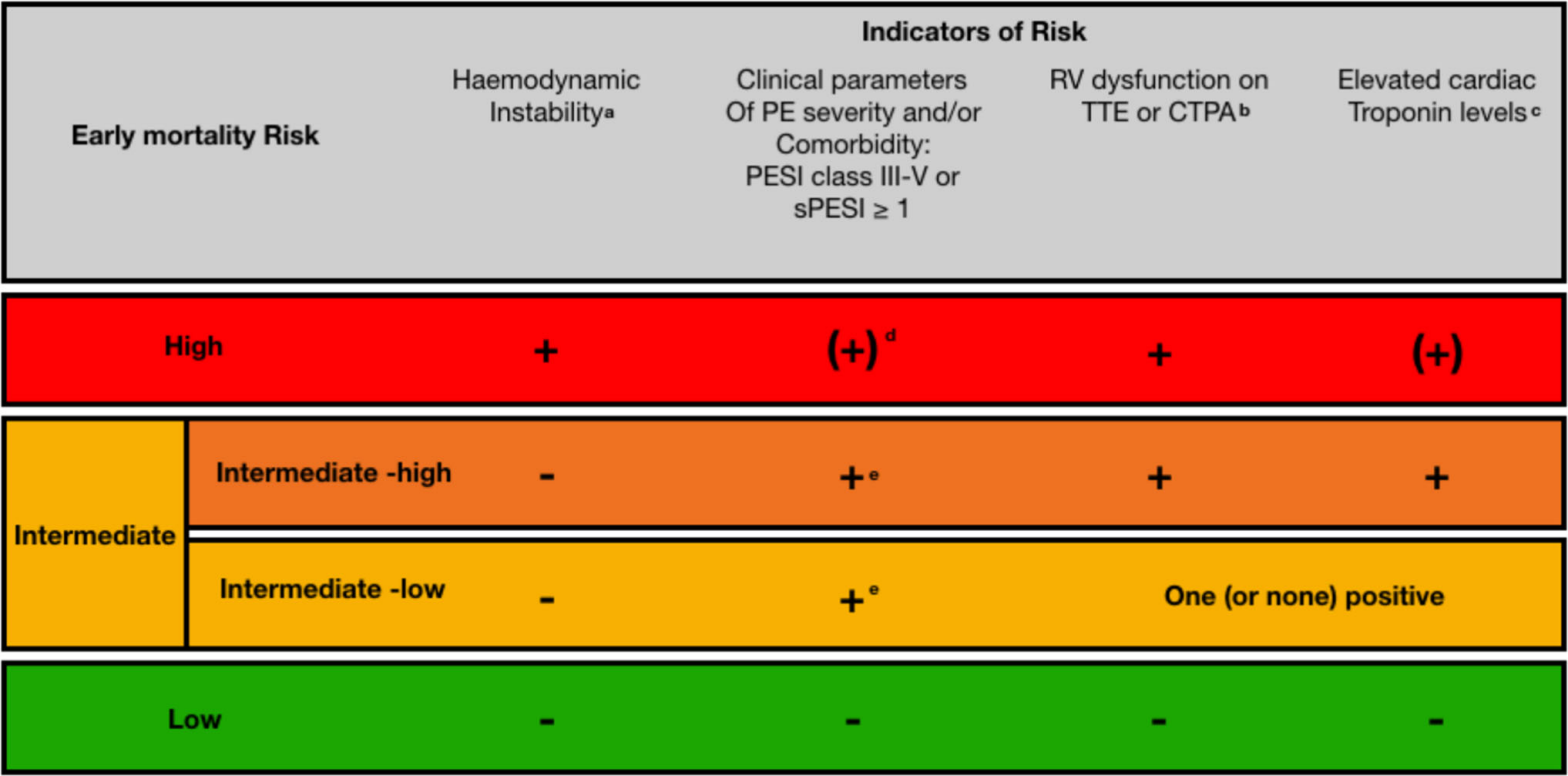
Stratification of early mortality risk in acute pulmonary embolism (PE) based on the 2019 ESC Guidelines. Haemodynamic instability indicates high-risk PE. Patients without instability but with adverse clinical parameters (e.g. PESI class III–V or sPESI ≥ 1) and/or evidence of RV dysfunction on TTE or CTPA, plus elevated cardiac troponin, are classified as intermediate–high risk. Low-risk PE is characterised by absence of all these markers. Adapted from the 2019 ESC Guidelines for the diagnosis and management of acute PE [3]. *PE* Pulmonary embolism; *PESI* Pulmonary Embolism Severity Index; *sPESI* simplified PESI; *TTE* Transthoracic echocardiogram; *CTPA* Computed tomography pulmonary angiography; *RV* Right ventricle; *LV* Left ventricle; *BNP* B-type natriuretic peptide; *NT-proBNP* N-terminal pro-BNP. ^**a**^ PESI class III–V or sPESI ≥ 1 indicates moderate to very high 30-day mortality risk or significant comorbidities. ^**b**^ Signs of RV dysfunction can be detected on TTE or CTPA (e.g. RV dilation, hypokinesis, or increased RV/LV ratio). (1) Right ventricular end-diastolic diameter > 30mm. (2) Right to left ventricular end-diastolic diameter > 0.9 (apical or subcostal four-chamber view). (3) Hypokinesis of right ventricular free wall (any view). (4) Tricuspid systolic velocity > 2.6 m/s (apical or subcostal four-chamber view). (5) If ECHO not available, CT imaging used to calculate right to left ventricular internal diameter ratio (a ratio > 0.9 denotes RV enlargement and RVD). ^**c**^ Elevated cardiac biomarkers (e.g. troponin, BNP, or NT-proBNP) indicate myocardial stress or injury. Myocardial injury confirmed by positive Troponin I (> 0.06 µg/L) or Troponin T (> 0.01 µg/L). ^**d**^ In the intermediate–high-risk group, both RV dysfunction and biomarker elevation should be present in addition to a positive clinical risk score. ^**e**^ Biomarker assessment may be optional; if measured and found negative, the patient remains at low risk

Patients should be risk-stratified according to the European Society of Cardiology (ESC) guidelines to help triage patients [3] (Fig. 2). Systemic anticoagulation is universally accepted as the standard of care in treating APTE [1]. Significant complications may occur, including death, primarily due to systemic thrombolysis and intracranial haemorrhage, with an incidence of intracranial haemorrhage reported at up to 3% [13, 14]. For high-risk and intermediate–high-risk patients, adjunctive therapeutic interventions are frequently indicated. Conventional management strategies include systemic thrombolytic therapy and/or surgical intervention.

Endovascular therapies are increasingly utilised, including catheter-directed thrombolysis (CDT), mechanical thrombectomy (MT). These interventions are used for patients with contraindications to systemic fibrinolysis or in cases where systemic thrombolytic therapy has proven ineffective. These procedures have demonstrated considerable efficacy with minimal major complications [3, 15–20]. Optimal therapeutic outcomes are observed when intervention is initiated within 48 h of symptom onset; however, clinical benefit may still be achieved in patients presenting with symptoms of up to 14 days duration [3].

### Indications and Contraindications for Endovascular Treatment of Acute Pulmonary Thromboembolism

#### Absolute Indications

1. Haemodynamically unstable patients in whom systemic anticoagulation and or fibrinolysis is contraindicated (such as previous surgery, high risk of bleeding, allergies) (evidence IIa C) [3].

2. Unstable patients in whom systemic fibrinolysis has failed (evidence IIa C) [3].

3. Patients with intermediate–high-risk (progressive clinical and echocardiographic deterioration) (evidence IIa C) [3].

4. Endovascular treatment may be considered if a thrombus is located in the proximal arteries (main and lobar arteries) as detected on CTPA, provided the patient is stratified as intermediate–high risk. However, if CTPA reveals a segmental thrombus only, an endovascular procedure is not indicated [17].

#### ***Relative Indication***s

1. Intermediate–high-risk patients, specifically haemodynamically stable patients who exhibit (a) RV dysfunction on echocardiography or CTPA and (b) myocardial injury as indicated by elevated laboratory biomarkers on admission. *Pulmonary Embolism Severity Index* (*PESI) Class III-IV or* a simplified PESI (*sPESI)* >  = *1* [3].

#### Contraindications

1. Patients with low-risk or intermediate–low-risk APTE.

#### Relative Contraindications

1. Allergy to iodinated contrast.

2. Kidney failure: defined by a creatinine clearance under 30 ml/min.

3. Subacute PE with symptoms > 14 days.

4. Inability to obtain consent.

## Patient Preparation

Pre-procedural planning includes a comprehensive assessment of the patient’s clinical status by anaesthesia/critical care doctors, ideally this should be as part of the PERT and the risk–benefit assessment of APTE (Fig. 2). Standard baseline blood tests including haemoglobin, electrolytes, clotting and cardiac biomarkers (see Table 1) should be reviewed along with relevant imaging and intravenous access should be obtained. Full anticoagulation as a background treatment for APTE must rapidly reach an activated partial thromboplastin time (aPTT) of 60–85 s or a ratio of 1.8–2.5, ideally with unfractionated heparin (UFH) therapy. The recommended UFH dosage is 80 IU/kg, followed by a continuous infusion of 18 IU/kg per hour [21]. If the patient has already received low-molecular-weight heparin (LMWH), the initiation of intravenous UFH must be delayed by 12 h. It should be taken into account that, in some cases, the patient may have received systemic fibrinolytic treatment, which increases the risk of systemic bleeding.

### Equipment

A high-quality digital subtraction fluoroscopy room is required as is ultrasound for puncture guidance. Access to gases for general anaesthesia should be available when necessary. Standard monitoring equipment should be available, including pulse BP, ECG and gases.

Standard equipment will vary depending on the type of endovascular treatment including the following:

o Access sheaths ranging from 5–26F 11–45 cm long.
o Guidewires 0.035´´ standard, hydrophilic and super stiff in a range of lengths, 145–260 cm.
o Catheters, i.e. cobra, Berenstain, pigtail, angled pigtail catheter, Swan Ganz catheter, thrombolytic catheters with multiple sideholes, EKOS systems, etc.
o Appropriate mechanical devices with which the operator is familiar.
o Appropriate thrombolytic agents, i.e. recombinant tissue plasminogen activator (rTPA) or urokinase.

## Treatment

The CIRSE checklist should be completed prior to commencement of the procedure.

### Monitoring

Continuous cardiac and respiratory monitoring is necessary for patients. Often procedures are performed under local anaesthesia. Depending on the patient’s clinical status, sedation or general anaesthesia with mechanical ventilation may be required.

Propofol use during catheter-directed interventions in patients with intermediate-risk APTE is linked to a higher rate of major adverse events compared to other sedatives like midazolam with fentanyl or morphine. Two studies reported high complication rate related to the use of Propofol, and therefore, it should be avoided for intraprocedural sedation in these patients due to the increased risk of complications, including the need for intubation, haemodynamic decompensation and in-hospital death (22, 23).

Extracorporeal membrane oxygenation (ECMO) or venoarterial extracorporeal membrane oxygenation (V-A ECMO) must be considered, in combination with endovascular treatment, for patients with APTE and refractory circulatory collapse or cardiac arrest [1, 3].

### Techniques

Vascular access is obtained via either the common femoral vein or the right internal jugular vein. The appropriate sheath introducer size is placed depending on the catheter or device to be used. Depending on the patient’s previous anticoagulation status, a bolus of 3–5000 units of UFH heparin should be given at this stage.

PA catheterisation is performed with an appropriate selective catheter (i.e. angled pigtail) to avoid damaging a papillary muscle, chordae tendineae or tricuspid valve [17, 19, 20]. Baseline direct transcatheter pulmonary pressure measurement should be performed before initiating thrombolysis or aspiration (normal pulmonary artery pressure (PAP) is below 20 mmHg, mean 12 mmHg) [17, 20].

Review of the CTPA will help identify the target PA for endovascular treatment. Depending on the patient’s pulmonary pressures and need for accurate pulmonary vasculature assessment, large volume and high-pressure injections should be minimised. Contrast can be manually injected in small quantities (10–20 ml bolus) to confirm the findings of the CTPA and aid catheter/device placement. If non-selective angiography is required, a pigtail catheter should be placed in the main pulmonary artery (PA), and a flow rate of approximately 8–10 mLs/s and a total volume of 15–20 ml [19, 20] has been recommended, although higher flow rates and volumes may sometimes be necessary. Digital subtraction imaging should be performed at 2–6 frames per second depending on the need to assess anatomy and disease extent or catheter positioning. Selective right and left lung angiographic studies will better delineate anatomy and clot burden.

- The right PA is best evaluated using a right anterior oblique (RAO) 30° projection.

- The left PA is best assessed with a left anterior oblique (LAO) 40° projection, allowing for a more accurate determination of thrombus extent and pulmonary perfusion.

Inferior vena cava (IVC) filters should be considered in patients with APTE and absolute contraindications to anticoagulation, and in cases of recurrence despite therapeutic anticoagulation (IIa C) [3, 24]. When employing a jugular approach, it is feasible to insert an IVC filter immediately prior to the pulmonary procedure. Conversely, when using a femoral approach, it is advisable to place the IVC filter after completing the pulmonary intervention.

### Specific Procedures

The following are commonly used endovascular techniques at the time of writing.

- Catheter-directed thrombolysis (CDT)

Ultrasound-guided puncture should be carried out into the jugular or femoral vein as appropriate. A 5 or 6F sheath should be placed into the access vein and selective pulmonary catheterisation should be undertaken with a selective catheter and hydrophilic guidewire to target either the PA with the most significant thrombus burden first or both PA using bilateral femoral access. Initially, fragment the thrombus by placing a pigtail catheter over the wire into the clot and manually rotate the preformed pigtail to increase the surface area of the thrombus, aiming to recanalise central vessels and enhance the effect of subsequent thrombolytics [20].

Insert a multi-side hole catheter (5 or 10 cm infusion length) into one or both PAs, as required, positioning the catheters within the thrombus. An alternative is to use a triple lumen sheath (Fast-Cath Trio, St Jude Medical, MN, US) which allows catheters to be placed in both PA with one venous access sheath.

In Europe rTPA (Alteplase or Tenecteplase) is most commonly used; however, urokinase may be used as an alternative agent [20].

Administer a bolus of 5 mg rTPA directly into the thrombus, followed by an infusion of 0.5–1 mg/hr of rTPA via each catheter (for up to 24 h with a total dosage below 30 mg)(25). Concurrently, the administration of a slow heparin infusion through the side port of the sheath to keep it patent could be considered (12 units/kg per hour, maximum 1000 units per hour).

Fibrinolysis may be combined with low-intensity ultrasound waves using a multi perforated catheter in an approach called ultrasound-assisted thrombolysis (UAT); this may reduce major bleeding complications and result in shorter thrombolysis periods and lower rTPA doses [26–28]. The patient should then be transferred to a high dependency or intensive care unit for close monitoring.

Patients should undergo clinical evaluation at 6, 12, 24 or 48 h, with monitoring of blood pressure, heart rate, oxygen saturation and laboratory markers including lactate, troponins, coagulation, and/or B-type natriuretic peptide (BNP) levels. Some studies also suggest PE monitoring [20].

The infusion typically lasts 6–24 h, depending on the protocol used, with a total average rTPA dose ranging from 10–30 mg. Systemic unfractionated heparin administration should be maintained throughout the endovascular fibrinolysis procedure, ensuring a Partial thromboplastin time (PTT) ratio of 1.5–2.5(25).

The infusion should be stopped when the patient achieves normalised blood pressure, clinical stability and commonly used clinical parameters, including:

Heart rate (HR) < 100 beats per minute, oxygen saturation > 95% or reduced supplemental oxygen requirement, respiration rate < 20 breaths per minute, improvement in RV/LV ratio on TEE or CTPA or Tricuspid Annular Plane Systolic Excursion (TAPSE) > 16 mm in transthoracic echocardiogram (TTE) and/or the improvement of mean Pulmonary artery pressure (mPAP) > 10 mmHg [20]. For intermediate–high-risk patients, at least 6 h of UAT or 12 h with a multiperforated catheter is recommended, with cessation of therapy when clinical parameters have normalised.

The rTPA infusion should be discontinued if fibrinogen levels reach < 200 mg/dL or drop below 50% of baseline levels or if a significant haemorrhagic complication occurs.

b) Mechanical thrombectomy (MT)

MT techniques include the use of large-bore aspiration catheters, pharmacomechanical catheters and computer-aided aspiration catheters. Often, MT is combined with CDT. Following ultrasound-guided jugular or femoral vein access, a large sheath is placed proportional to the chosen device. Longer sheaths are often necessary to support the device’s passage into the pulmonary artery and during thrombectomy placed over 0,035″ superstiff guidewires.

### Devices

The following are some of the more commonly used devices at the time of writing.

The Penumbra Indigo® device (Penumbra, Alameda, CA) is an aspiration thrombectomy catheter connected to a high-pressure suction pump. It is available in sizes ranging from 8 to 16 French. During suctioning, rapid blood loss may occur and should be monitored in the collecting chamber, as the current system does not allow recycling of aspirated blood. The catheter can become clogged or obstructed during the procedure. To address this, it is often used with a retractor guide (SEP®-separator-) to try to unclog it. If the retractor guide fails to unclog the catheter, the catheter should be removed and flushed with saline. Large thrombectomy catheters (16 French) do not include retractor guide. In addition, the computer-aided mechanical aspiration systems minimise blood loss [19, 20, 29, 30].

The AngioVac™ device (Angiodynamics, Latham, NY) is a large-bore aspiration device which can evacuate large clot loads while recirculating filtered blood. The device requires placement of a large-calibre sheath (26 and 16Fr) and general anaesthesia, along with the support of a perfusionist while the patient is placed on extracorporeal veno-venous bypass. The suction catheter is rigid and can be challenging to navigate safely in the pulmonary territory [31].

The Inari™ FlowTriever that have three components (nitinol discs or a laser-cut open-cell nitinol disc, a large-bore Triever aspiration catheter and a proximal aspiration/retraction handle). The first step is aspiration of proximal clots; then the inner wire shape (composed of three softly braided nitinol discs or a new laser-cut open-cell nitinol disc) is unsheathed into peripheral and smaller thrombosed target vessels. The discs are designed to fit and disrupt the clot without damaging the vessel walls. Once thrombus is captured, retraction is done through the aspirator handle, which combines aspiration forces and mechanical retraction to remove the clot-containing nitinol discs through the large-bore catheter. The system optionally incorporates the FlowSaver® Blood Return System, which filters aspirated blood through a 40-µm dual-layer polyester filter (with < 0.1% haemolysis) to reinfuse autologous blood intra-procedurally, reducing blood loss to under 100 mL. The device comes in three sizes that can be selected depending on the size of the target vessel (16, 20 and 24Fr). To accommodate the relatively large introducer sheath, an incision at the venotomy site is required. As with all mechanical or aspiration devices, these can be used with or without local thrombolytic injection. The vascular access site requires closure with a closure device or a purse string suture [32].

## Post-Treatment Follow-up Care

The patient should be transferred to a critical care unit for monitoring after CDT or MT until stable and able to return to a regular ward. Systemic anticoagulation, as previously agreed upon by the multidisciplinary team (either therapeutic UFH infusion or LMWH), should continue until the patient is switched to oral treatment (direct oral anticoagulants) before discharge. The patient must maintain a therapeutic range of UFH until they transition to LMWH or direct oral anticoagulants (DOACs) for at least for 3/6 months [33].

Follow-up echocardiography or CTPA should be performed within 24–48 h of the intervention to assess change in RV parameters. After discharge, clinical follow-up is recommended, including ongoing anticoagulation management. If a IVC has been placed, its removal must be scheduled in the IR department within the manufacturers’ instruction, or earlier if anticoagulation has been re-introduced. If patients present with new-onset symptoms, a CTPA should be considered to evaluate for APTE recurrence.

## Outcomes

Technical success of APTE interventions such as CDT or MT is usually defined by a reduction > 30–50% in the thrombotic burden (Qanadli score) in the main or lobar PA [34]. A technical success of > 90% [3, 17, 19, 35, 36] is reported for endovascular techniques. Clinical success is variously defined by a patient’s haemodynamic stabilisation, decrease in pulmonary hypertension (PH) and RV overload and increased partial pressure of oxygen; these factors are prognostic indicators that influence the survival rate in patients with intermediate or high-risk APTE [16, 17, 19]. Clinical success rates > 80% (Table 2) are reported. Possible reasons for treatment failure include the development of irreversible right-sided heart strain and cardiogenic shock prior to the initiation of treatment. Additionally, the presence of a chronic PE component not amenable to catheter interventions can contribute to treatment failure [19, 20].

**Table 2:** Review of clinical trial data on acute pulmonary thromboembolism therapies

Clinical trial	N	Treatment	Randomisation and comparison	Follow-up time (days)	Intermediate risk PE /Submassive PE n (%)	High-risk PE/Massive n(%)	Deaths / Major Bleeding	Efficacy outcomes
PERFECT [17]	101	Tissue plasminogen activator (tPA) or urokinase. Catheter-directed	No randomisation Single arm	30	73 (72%)	28 (28%)	6/101 (5.9%) 0/101(0%)	PASP
ULTIMA [24]	59	tPA (10–20 mg) + USAT n = 30 USAT vs n = 29 Heparin	Randomisation Heparin	90	59 (100%)	0	USAT 0/30 (0%); 0/30 (0%) Heparin 1/29 (3.44%); 0/29(0%)	Reduction of RV/LV ratio
SEATLE II [25]	150	tPA (24 mg) + USAT	No randomisation Single arm	30	119 (79%)	31 (21%)	4/150 (2.6%); 17/150(11.3%)	Reduction of RV/LV ratio PASP
OPTALYSE PE [26]	101	tPA (8–24 mg) + USAT	Randomisation with 4 different protocols with tPA	365	101 (100%)	0	2/101(1.98%); 4/101 (3.96%)	Reduction of RV/LV ratio
KNOCOUT PE [31]	489	tPA (mean 18.1 mg) + USAT	No randomisation	365	463 (94.7%)	26 (5.3%)	1.02% (5/489); 1.6% (8/489)	Reduction of RV/LV ratio TAPSE RVSP PEmb-QoL & EQ-ED-5L
FLARE, 2019 [30]	106	FlowTriever® Inari Pulmonary thrombectomy	No randomisation Prospective registry	30	104 (100%)	0		Reduction of RV/LV ratio
FLASH [32]	800	FlowTriever® Inari Pulmonary thrombectomy	No randomisation	180	737 (92.1%)	64 (7.9%)	0.75% (6/800); 1.4% (11/800)	Reduction mPAP Increase CI
FLAME [33]	115	FlowTriever® Inari pulmonary thrombectomy (n = 53) vs Context treatment (Anticoagulation ± tPA) (n = 61)	No randomisation parallel group Observational study FlowTriever vs Context	45	0	115 53 FlowTriever vs 61 Context (tPA + Anticoagulation)	FlowTriever ARM 1/53 (1,9%); 6/53 (11.3%) Context ARM 18/61(29.5%); 15/61 (24.6%)	in-hospital composite of all-cause mortality, bailout to an alternate thrombus removal strategy, clinical deterioration and major bleeding
PEERLESS I [34]	550	FlowTriever® Inari Pulmonary thrombectomy (274) vs CDT ± USAT (276)	Randomised 1:1 LBMT vs CDT	30	550	0	LBMT 19/274 (6.9%); vs CDT 21/276 (7.60%)	Hierarchal win ratio 1.) all-cause mortality, 2.) ICH, 3.) major bleeding 4.) clinical deterioration 5.) postprocedural intensive care unit (ICU)
EXTRACT-PE [28]	119	Indigo/penumbra 8Fr Pulmonary thrombectomy	No randomisation Single arm	30	118	1		Reduction ratio VD/VI
STRIKE-PE (Interim Analysis) [27]	150	Aspiration thrombectomy 12FR CAVT	No randomisation Single arm	365	142/150 (94.6%)	8/150 (5.33%)	3/150 (2.0%); 2/150 (1.3%)	Reduction of RV/LV ratio PASP mPAP

*CAVT* Computer Assisted Vacuum Thrombectomy; *CDT* Catheter-directed thrombolysis*; CI* Cardiac Index < 2L/min/m^2^, *ICH* Intracranial Haemorrhage; *LV* Left Ventricle; *mPAP* Mean Pulmonary Artery Pressure; *PASP* Pulmonary Artery Systolic Pressure; *PE* Pulmonary Embolism; *PEmb-QoL* Pulmonary Embolism Quality of Life Questionnaire; *RV* Right Ventricle; *RV/LV* Right ventricle/left ventricle ratio*; RVSP* Right Ventricular Systolic Pressure; *TAPSE* Tricuspid Annular Plane Systolic Excursion; *tPA* Tissue Plasminogen Activator; *USAT* Ultrasound Assisted Thrombolysis

The incidence of major bleeding complications is lower with CDT compared to systemic thrombolysis [29, 37, 38]. A recent prospective, single-arm and multicentre trial demonstrated the efficacy and safety related to a specific MT system with a major complication rate of 3.8% and the need for adjunctive thrombolytics in only 2% of patients [31]. Significant complications have been reported including perforation of the RV free wall and embolisation of distal clots [30]. Reported intraprocedural mortality in CDT and MT procedures is < 1% [29, 38].

## Conclusion

Endovascular treatments in APTE are being increasingly utilised and the range as well as sophistication of treatments is rapidly evolving. Multi-detector CTPA is the primary investigation for diagnosing APTE and intravenous fibrinolysis is still considered first-line treatment. However, in cases where fibrinolysis is contraindicated, failed and there is haemodynamic instability, CDT and/or MT should be utilised, which have a high reported technical and clinical success.

## Abbreviations

APTE: Acute pulmonary thrombo-embolism
aPTT: Activated partial thromboplastin time
BNP: B-type natriuretic peptide
CDT: Catheter-directed thrombolysis
CI: Cardiac Index
CPTEH: Chronic pulmonary thromboembolic hypertension
CPTED: Chronic pulmonary thromboembolic disease
DOAC: Direct oral anticoagulant
DVT: Deep vein thrombosis
ECMO: Extracorporeal membrane oxygenation
IVC: Inferior vena cava
LAO: Left anterior oblique
LMWH: Low-molecular-weight heparin
mPAP: Mean pulmonary artery pressure
MT: Mechanical thrombectomy
NT-proBNP: N-terminal pro-BNP
NYHA: New York Heart Association
PA: Pulmonary artery
PAP: Pulmonary artery pressure
PE: Pulmonary embolism
PEmb-Qo: Pulmonary Embolism Quality of Life Questionnaire
PERT: Pulmonary Embolism Response Team
PESI: Pulmonary Embolism Severity Index
PH: Pulmonary hypertension
PVR: Pulmonary vascular resistance
RAO: Right anterior oblique
RHC: Right heart catheterisation
rTPA: Recombinant tissue plasminogen activator
RV: Right ventricular
RV/LV: Right ventricle/left ventricle ratio
SOP: Standards of Practice
sPESI: Simplified Pulmonary Embolism Severity Index
TAPSE: Tricuspid Annular Plane Systolic Excursion
TEE: Transoesophageal echocardiogram
tPA: Tissue plasminogen activator
TTE: Transthoracic echocardiography
UAT: Ultrasound-assisted thrombolysis
UFH: Unfractionated heparin
V-A ECMO: Venoarterial extracorporeal membrane oxygenation
V/Q: Ventilation/quantity of perfusion scan
VTE: Venous thromboembolic disease
